# Fission Yeast Sec3 and Exo70 Are Transported on Actin Cables and Localize the Exocyst Complex to Cell Poles

**DOI:** 10.1371/journal.pone.0040248

**Published:** 2012-06-29

**Authors:** Felipe O. Bendezú, Vincent Vincenzetti, Sophie G. Martin

**Affiliations:** Department of Fundamental Microbiology, Faculty of Biology and Medicine, University of Lausanne, Lausanne, Switzerland; Institute of Developmental Biology and Cancer Research, France

## Abstract

The exocyst complex is essential for many exocytic events, by tethering vesicles at the plasma membrane for fusion. In fission yeast, polarized exocytosis for growth relies on the combined action of the exocyst at cell poles and myosin-driven transport along actin cables. We report here the identification of fission yeast *Schizosaccharomyces pombe* Sec3 protein, which we identified through sequence homology of its PH-like domain. Like other exocyst subunits, *sec3* is required for secretion and cell division. Cells deleted for *sec3* are only conditionally lethal and can proliferate when osmotically stabilized. Sec3 is redundant with Exo70 for viability and for the localization of other exocyst subunits, suggesting these components act as exocyst tethers at the plasma membrane. Consistently, Sec3 localizes to zones of growth independently of other exocyst subunits but depends on PIP_2_ and functional Cdc42. FRAP analysis shows that Sec3, like all other exocyst subunits, localizes to cell poles largely independently of the actin cytoskeleton. However, we show that Sec3, Exo70 and Sec5 are transported by the myosin V Myo52 along actin cables. These data suggest that the exocyst holocomplex, including Sec3 and Exo70, is present on exocytic vesicles, which can reach cell poles by either myosin-driven transport or random walk.

## Introduction

Polarized exocytosis is a fundamental biological process critical for very diverse functions, such as cell migration, polarized growth and cell-cell signaling [1], [2]. Polarized exocytosis relies on the directed transport of exocytic vesicles along cytoskeletal networks and on their local tethering and fusion with the plasma membrane. The budding yeast *Saccharomyces cerevisiae* model system has played an invaluable role in identifying many of the players and elucidating their role in polarized exocytosis [3]. In this system, the master polarity regulator Cdc42 is activated at incipient bud sites and serves to activate members of the formin family of actin nucleators to assemble polarized arrays of actin cables [4], [5], [6], [7]. These cables serve as tracks for the type V myosin mediated delivery of secretory vesicles to sites of growth [8]. At the plasma membrane, secretory vesicles are tethered by the multi-subunit exocyst complex, which is also activated by Cdc42, leading to SNARE-mediated fusion of vesicles [9], [10].

The exocyst is a universally conserved eukaryotic multi-subunit complex composed of eight members: Sec3, Sec5, Sec6, Sec8, Sec10, Sec15, Exo70 and Exo84. This complex was discovered in budding yeast, a system that has since contributed greatly to our understanding of its function and regulation [11], [12]. The exocyst plays central roles in many exocytic events in all cell types: for instance it is required for polarized growth, cell division, directional migration or glucose transporter localization [9]. A prevalent model for exocyst function states that its primary role is to provide spatio-temporal information for the recruitment and tethering of Golgi derived secretory vesicles [13], [14]. Its role as a tethering factor is supported by EM analysis of exocyst holocomplex and crystal structures of several members [15]. Although at the amino acid sequence level exocyst members are poorly conserved, they adapt similar helical bundles, forming a holocomplex composed of side-by-side interacting helixes [16], [17], [18], [19], [20].

Structure studies have corroborated many genetic and biochemical descriptions of protein-protein and protein-lipid interactions. The emerging picture from these studies finds that Sec3 and Exo70 provide positional information for localization of the entire complex at the bud tip by binding both Rho proteins and phosphatidylinositol 4,5-bisphosphate (PIP_2_). Both Sec3 and Exo70 interact with PIP_2_ and the crystal structure of N-terminal domain from budding yeast Sec3 revealed a cryptic PIP_2_-binding pleckstrin homology (PH) domain [21], [22], [23], [24]. Sec3 and Exo70 are also direct effectors of Cdc42, Rho1 and Rho3, all of which localize to sites of growth and, along with PIP_2_, provide spatial determination for exocyst localization [22], [25], [26], [27]. In contrast, the remaining exocyst members require myosin V- mediated transport along actin cables for localization to sites of growth, with Exo70 also transported in this way [28]. Domain analysis has found that budding yeast requires at least one domain from either Sec3 or Exo70 capable of binding Cdc42 and PIP_2_ for viability, underscoring the importance of these two proteins as spatial landmarks for polarized exocytosis [22].

The fission yeast, *Schizosaccharomyces pombe*, grows by polar extension at cell tips and divides by medial fission. In this organism, the exocyst is essential for cell division [29] and contributes to polarized cell growth [30], [31], [32]. For polarized growth, actin cables are assembled from the poles of the cell by the formin For3 and used by the myosin V Myo52 to transport cell wall remodeling enzymes-containing vesicles to cell poles [33], [34], [35], [36], [37]. However, actin cables and type V myosins are dispensable for polarized exocytosis because the exocyst complex does not require actin based transport to localize to cell tips [30], [32]. In absence of vesicle transport, cells rely entirely on the exocyst to mediate the capture of secretory vesicles at the sites of growth. Thus, myosin-driven transport and the exocyst play complementary roles for polarized exocytosis in fission yeast [30], [31], [32].

Two features can be noted in our current understanding of the exocyst in fission yeast. First is the absence of a characterized Sec3 homologue. Second, Exo70 is not essential for growth at moderate temperatures; *exo70*Δ cells maintain wildtype shape and growth rate when grown at 25°C [38]. It is thus unclear whether the role of Sec3 and Exo70 as landmarks for delivery of other exocyst subunits is conserved in this organism.

Here we describe the identification and characterization of Sec3 in fission yeast. We show Sec3 and Exo70 are together essential for spatially determining exocyst localization. Although all the exocyst subunits examined localize to cell tips largely independently of the actin cytoskeleton, all including Sec3 are normally transported to cell tips by the myosin V Myo52. These findings suggest Sec3 is present on exocytic vesicles.

## Results

### Identification of *S. pombe* Sec3

**Figure 1:**
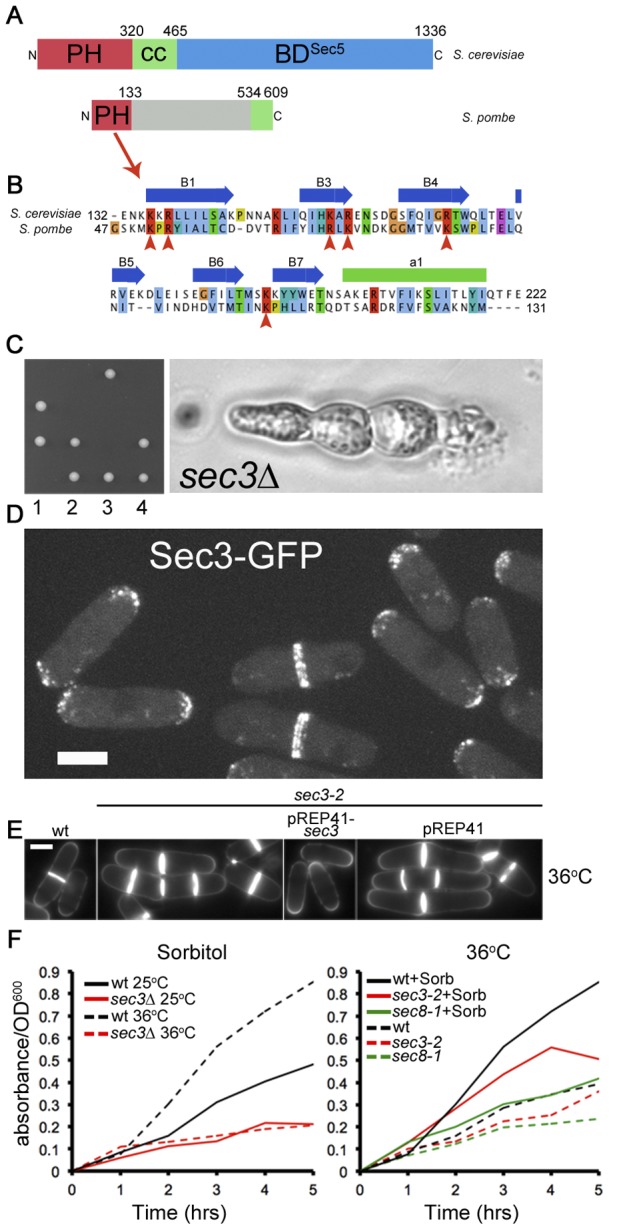
Function and localization of *S. pombe* Sec3. **A.** Scheme of Sec3 in *S. cere*visiae and *S. pombe*. Cryptic PH domains are shown in red, predicted coils in green. The C-terminal Sec5-binding region of *S. cerevisiae* Sec3 [25] is not conserved in *S. pombe*. **B.** Alignment of Sec3 PH domain. Critical residues predicted to contact phospholipids are indicated with red arrowheads and are conserved in *S. pombe*. **C.** Tetrad dissection of *sec3*Δ*::kanMX/sec3+* diploids on YE plate and terminal multi-septated phenotype of unviable spore. None of the viable spores grew on G418 plates (not shown). **D.** Maximum projection of spinning disk confocal sections of Sec3-GFP. Bar is 5 µm. **E.** Calcofluor-stained wildtype and *sec3-2* cells grown at 36°C for 6 h. Note that the multi-septated phenotype of *sec3-2* is rescued by plasmid-expression of *sec3+* (pREP41-sec3+), but not empty vector (pREP41). Bar is 5 µm. **F.** Secretion of acid phosphatase in wildtype and *sec3* mutants. Left: wildtype and *sec3*Δ mutants were pre-grown in EMM 1 M sorbitol at 25°C and kept at 25°C or shifted to 36°C at t = 0. Right: wildtype, *sec3-2* and *sec8-1* mutants were pre-grown at 25°C in EMM 1 M sorbitol and shifted to 36°C ± sorbitol at t = 0.

The N-terminal domain of *S. cerevisiae* Sec3 contains a cryptic pleckstrin homology (PH) fold [23], [24]. A homology search against *S. pombe* predicted proteins using this domain identified the *SPAC17G8.12* ORF as having a similar N terminal domain (Figure 1A). Importantly, all six positively charged lysine and arginine residues in the PH domain predicted to bind the PIP_2_ head group are conserved in this candidate (Figure 1B). If *SPAC17G8.12* encodes the missing Sec3 protein in fission yeast, we expected its null phenotype and sub-cellular localization to be similar to other exocyst members. Sporulation of heterozygous mutant *SPAC17G8.12+/SPAC17G8.12*Δ diploid on standard growth medium gave rise to 2 viable and 2 lethal progeny that exhibited a terminal phenotype of elongated muti-septated cells (Figure 1C), identical to most other exocyst mutants, *exo70*Δ excluded [29], [38]. C-terminal tagging at its chromosomal locus with GFP revealed an exocyst-like localization at both the cell tips and in a double ring at the site of cell constriction (Figure 1D). We thus named the *SPAC17G8.12* gene *sec3*.

To facilitate further experiments, we generated a temperature-sensitive lethal allele, *sec3-2*, which again displayed multi-septated cells when grown at restrictive temperature (Figure 1E). Expression of plasmid-encoded *sec3* rescued the temperature sensitive lethality and multi-septated phenotype of the *sec3-2* mutant (Figure 1E and data not shown). As final evidence, *sec3-2* mutants (as well as *sec3*Δ mutants, see below) were found to display defects in acid phosphatase secretion similar to the *sec8-1* mutant (Figure 1F) [29]. Taken together, we conclude *SPAC17G8.12* encodes the missing *sec3* gene. We note, however, that besides the PH domain there appears to be little similarity between the Sec3 proteins of *S. pombe* and *S. cerevisiae* (Figure 1A).

### Conditional Essentiality of Sec3 and Exo70

**Figure 2:**
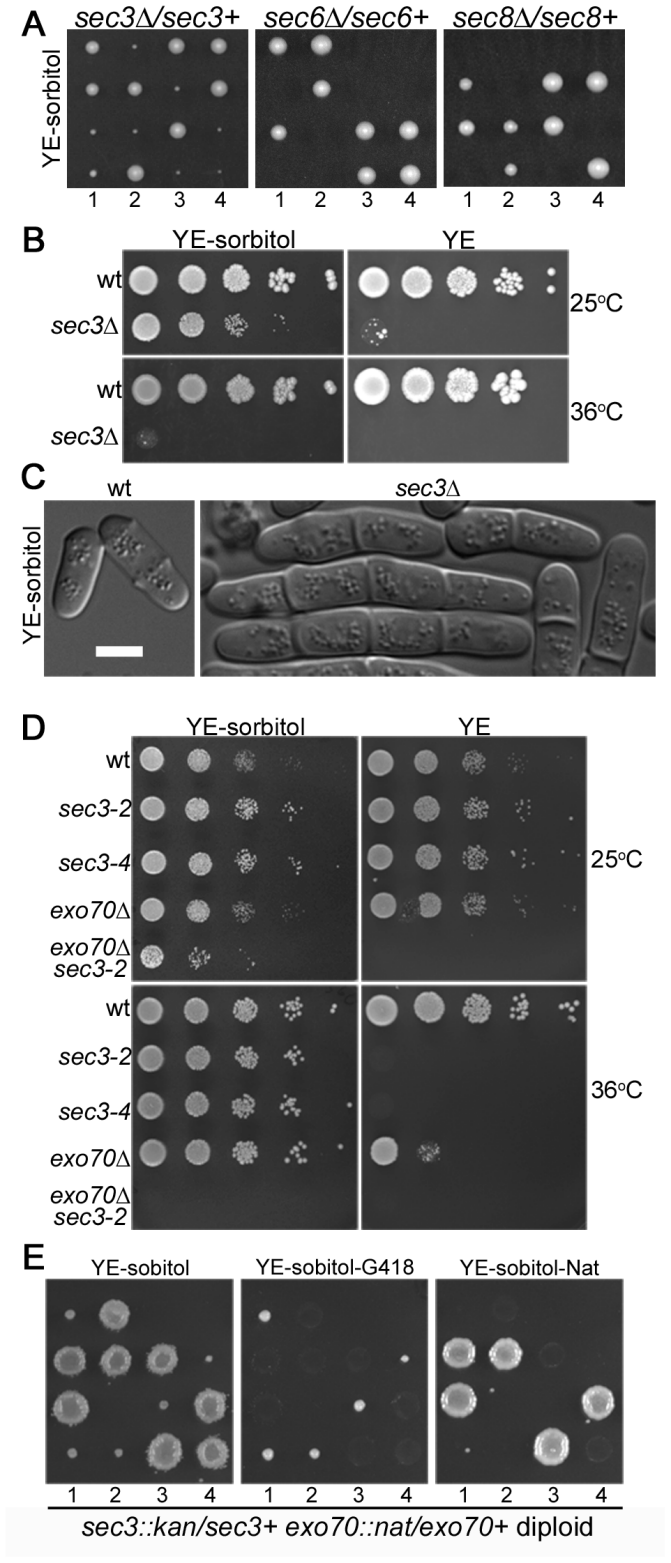
Synthetic lethality of *sec3* and *exo70*. **A.** *sec3*Δ, but not *sec6*Δ or *sec8*Δ, is viable in presence of 1 M sorbitol. Indicated diploids were sporulated and spores dissected on YE-sorbitol plates. Small colonies were *sec3*Δ, as they grew in presence of G418, while large colonies were wildtype as they failed to grow (not shown). **B.** 10-fold serial dilutions of indicated strains, showing *sec3*Δ grows in presence of 1 M sorbitol, but dies at 36°C. **C.** DIC images of wildtype and sec3Δ grown in YE-sorbitol at 25°C. Bar is 5 µm. **D.** 10-fold serial dilutions of indicated strains, showing *exo70*Δ *sec3-2* synthetic lethality. **E.** Tetrad dissection of *sec3*Δ*::kanMX/sec3+ exo70*Δ*::natMX/exo70+* diploid. Tetrad 1 shows a parental ditype, tetrads 2 to 4 are tetratypes, from which the double mutant spore is unviable, indicating synthetic lethality of *sec3*Δ and *exo70*Δ.

Exo70 is conditionally essential. When grown at 25°C *exo70*Δ cells lack any detectable growth or shape defects [38]. However, when grown at 36°C *exo70*Δ cells die with a typical exocyst-null terminal phenotype of elongated multi-septated cells [38]. Interestingly, the temperature sensitive lethality of the hypomorphic *sec8-1* mutant is rescued by the addition of the osmotic stabilizer sorbitol [39]. We found that 1 M sorbitol also suppressed *exo70*Δ temperature sensitivity. Furthermore, sorbitol rescued the lethality of *sec3*Δ null cells at 25°C indicating that Sec3 is only conditionally essential for growth in fission yeast (Figure 2A and B). However, viable *sec3*Δ cells grown with sorbitol were heavily multi-septated, indicating delay in cell separation, showed a drastic reduction in growth rate (Figure 2C), and were significantly impaired in acid phosphatase secretion (Figure 1F). Rescue by sorbitol is specific for *sec3*Δ and *exo70*Δ cells, *as* neither *sec6*Δ nor *sec8*Δ were rescued (Figure 2A). Because both Sec3 and Exo70 provide spatial cues for exocyst targeting in budding yeast, we asked whether in fission yeast Sec3 and Exo70 are redundant. A *sec3-2 exo70*Δ double mutant was dead in absence of sorbitol or at 36°C and significantly more sick than either single mutant in the permissive condition of 25°C + sorbitol (Figure 1D). More significantly, sporulation of a *sec3*Δ*/sec3+ exo70*Δ*/exo70+* diploid showed lethality of *sec3*Δ *exo70*Δ double mutant spores, which was not rescued by the addition of sorbitol (Figure 1E). These results indicate that fission yeast cells require either Sec3 or Exo70 for viability, with Sec3 playing a more important role as judged from the severity of its null phenotype. These results support the model that Sec3 and Exo70 play redundant functions, possibly by directing the remaining exocyst complex to the sites of growth.

### Either Sec3 or Exo70 is Necessary for Exocyst Localization

**Figure 3:**
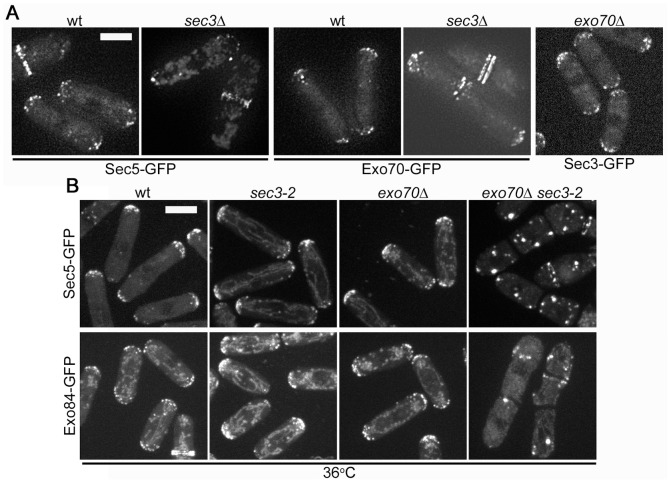
Sec3 and Exo70 are redundantly required for the localization of the exocyst to cell poles. **A.** Sec5-GFP, Exo70-GFP and Sec3-GFP localization in indicated genotypes at 25°C, showing Sec5 localizes to sites of growth in *sec3*Δ cells and Sec3 and Exo70 localize independently of each other. **B.** Localization of Sec5-GFP and Exo84-GFP in wildtype, *sec3-2*, *exo70*Δ and double mutants grown for 90 min at 36°C, showing loss of pole localization only in double mutants. Note that there is a weak residual localization to septated regions. All images are maximum projection of spinning disk confocal sections. Bars are 5 µm.

Fission yeast can thus live in the absence of either Sec3 or Exo70, but not both. Because the remaining exocyst members are all unconditionally essential, we reasoned that only one of the two is required to recruit the remaining members to the sites of growth to carry out their essential function. To test this idea, we first confirmed that Sec3-GFP or Exo70-GFP localized properly at the cell tips in the absence of the other (Figure 3A). In addition, Sec3-GFP localized normally to cell tips in *sec8-1* mutants (data not shown). The unconditional synthetic lethality of the *sec3*Δ *exo70*Δ double mutant did not allow us to determine the localization of exocyst members in the null mutants. We therefore localized Sec5-GFP or Exo84-GFP in the *sec3-2 exo70*Δ strain grown at 36°C. While both Sec5-GFP and Exo84-GFP localized to cell tips in either wt, *sec3-2* or *exo70*Δ single mutants at 36°C, both failed to localize properly in the *sec3-2 exo70*Δ double mutant grown at 36°C (Figure 3B). In fact, both Sec5-GFP and Exo84-GFP formed aggregates throughout the cytoplasm. Sec5-GFP was also able to localize to sites of growth and division in *sec3*Δ cells (Figure 3A). Thus either Sec3 or Exo70 is sufficient to provide the spatial landmarks for recruitment of other exocyst members to the cell tips. The localization of Sec5-GFP or Exo84-GFP in the absence of *sec3* and *exo70* may represent aggregated vesicles due to the loss of spatial landmarks.

### Sec3 Requires Cdc42 and PIP_2_ but not Actin Cables for Cell Tip Localization

**Figure 4:**
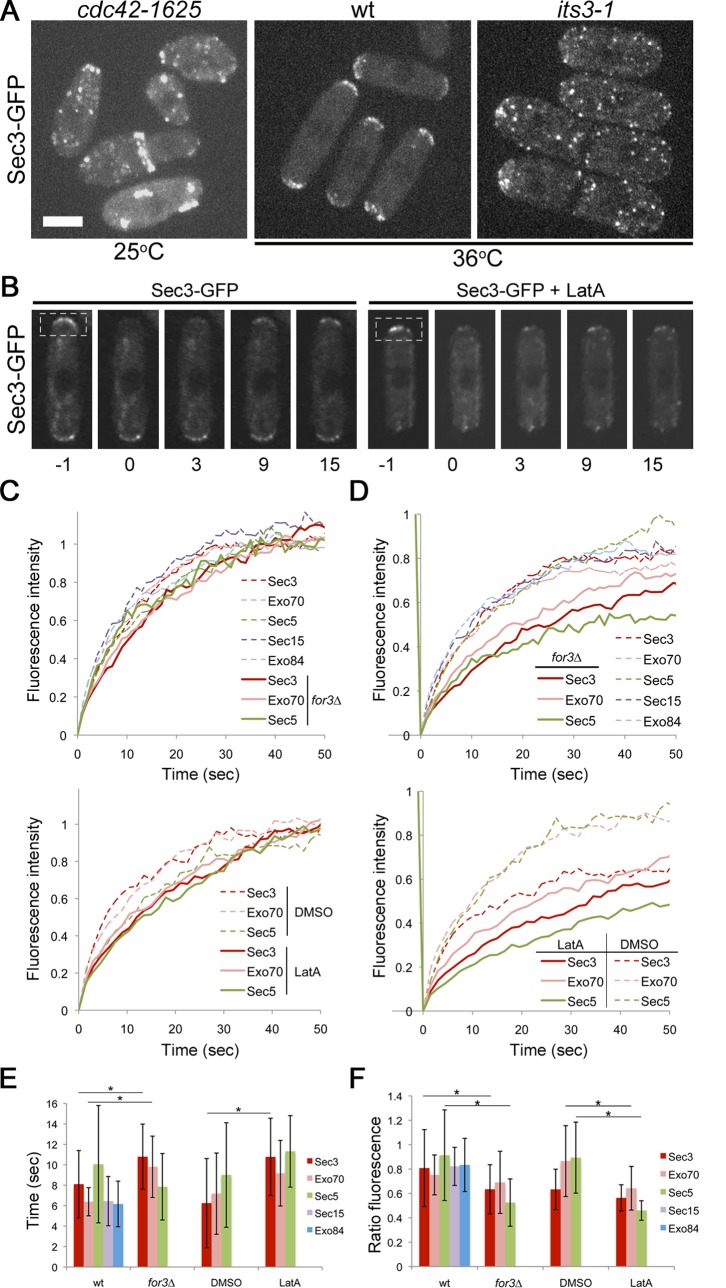
Sec3, like other exocyst subunits, localizes to cell poles in a Cdc42 and PIP_2_-dependent, but largely actin-independent manner. **A.** Maximum intensity projection of spinning disk confocal sections of Sec3-GFP in indicated genotypes. Bar is 5 µm. **B.** Timelapse of FRAP experiment of Sec3-GFP with or without 200 µM LatA. Indicated region was bleached. Time 0 was taken immediately post-bleach. Time is indicated in seconds. Single spinning disk confocal sections are shown. **C-D.** Average recovery of GFP-tagged exocyst subunits to bleached cell poles. Top: Untreated wildtype or *for3*Δ cells with indicated GFP-tagged subunits. Bottom: wildtype cells with indicated GFP-tagged subunits treated with 200 µM LatA or DMSO as control. The same data is shown in C and D. In C, all curves were normalized such that maximal recovery  =  1, to highlight similar recovery halftimes between 6 and 12 s for all curves. Note that *for 3*Δ and LatA curves globally show a tendency for slightly longer halftimes as compared to wildtype and untreated samples. In D, curves were normalized such that pre-bleach signal  =  1, to highlight the differences in mobile fractions. Note a tendency for reduced mobile fraction in *for3*Δ and LatA curves. For each condition, the curves show average of 16 or more bleached cell tips. **E-F.** Average recovery halftimes (E) and average mobile fraction ratio (F) for all strain analyzed in panels C-D. Error bars show the standard deviation of the mean. Significantly different values (unpaired Student’s t-test p<0.05) are indicated by asterisks.

We have previously shown that Sec6 and Sec8 require active Cdc42 and PIP_2_ but not actin cables or type V myosins for localization to the cell tips [32]. We extended these analyses to the localization requirements of Sec3. Dependences on active Cdc42 and PIP_2_ were examined by localizing Sec3-GFP in the *cdc42-1625* and the *its3-1* mutant, respectively. The *cdc42-1625* allele results in drastic reduction of activity even at 25°C without compromising cell tip localization [32], [40]. The *its3-1* allele is a temperature sensitive mutation in the major phosphatidylinositol 4-phosphate 5-kinase and leads to greatly reduced levels of PIP_2_ when cells are grown at 36°C [41]. Similar to Sec6 and Sec8, Sec3-GFP showed a drastic reduction in localization at the cell tips in *cdc42-1625* cells and near loss of tip localization in *its3-1* cells grown at 36°C (Figure 4A). Thus, consistent with other exocyst proteins, Sec3 requires active Cdc42 and PIP_2_ for proper tip localization.

We also investigated the localization dynamics of Sec3-GFP and other exocyst subunits at the cell tips by FRAP (fluorescence recovery after photobleaching) analysis (Figure 4B). Following photobleaching of Sec3-GFP signal from the cell tips Sec3-GFP recovery dynamics was nearly identical to those of Sec5-, Sec15-, Exo70-, Exo84-, Sec6- and Sec8-GFP, all displaying a half-time of recovery between 6 and 10 seconds (Figure 4C and E) [32]. Recovery dynamics was only mildly affected for Sec3-, Sec5- and Exo70-GFP in a *for3*Δ mutant background, which lacks actin cables, or following actin depolymerization by treatment with a high concentration (200 µM) of the F-actin inhibitory drug LatA (Figure 4C and E) with half-times of recovery between 8 and 11 seconds, similar to Sec6- and Sec8-GFP [32]. As for Sec6 and Sec8, the mobile fraction was reduced upon LatA treatment [32] (Figure 4D), suggesting that actin-based processes contribute, though moderately, to efficient recycling of the exocyst to the cell tips (Figure 4D and F). We note that for Sec3, treatment with the DMSO solvent control alone also caused moderate changes in the mobile fraction. In *for3*Δ cells, there was also a tendency for smaller mobile fractions (Figure 4D and F). Pairwise comparisons showed some of these differences in halftime and mobile fraction in cells with intact or disrupted actin cytoskeleton were significant (Figure 4E-F). Thus all exocyst subunits analyzed to date share similar requirements for cell tip localization. All show dependence on active Cdc42 and PIP_2_ but depend only moderately on F-actin. Furthermore, similar dynamics at the cell tips suggest that all exocyst members may exist preassembled on secretory vesicles prior to recruitment to the cell tips.

### Myo52 Transports Exocyst Members to Cell Tips along Actin Cables

**Figure 5:**
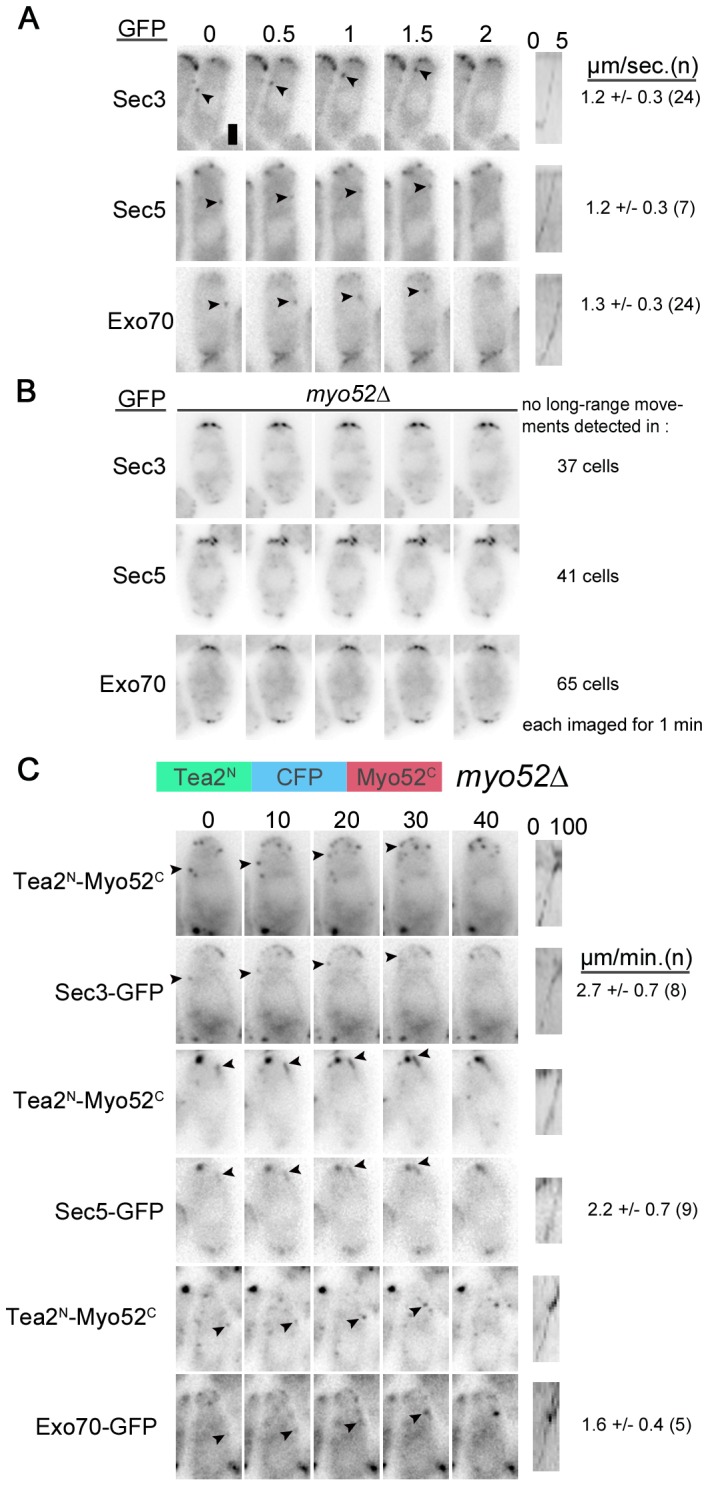
Sec3, Exo70 and Sec5 are transported towards cell poles by myosin V Myo52. **A-B.** Timelapse images of Sec3-, Sec5- and Exo70-GFP in wildtype (A) and *myo52*Δ cells (B). Arrowheads point to moving dots. A kymograph along the path of the indicated dot is shown on the right. Average rate of movement is shown on the right. Time is indicated on top in seconds. **C.** Timelapse images of Sec3-, Sec5- and Exo70-GFP in *myo52*Δ cells expressing a Tea2^N^-CFP-Myo52^C^ chimera. GFP and CFP signals are shown. Arrowheads point to moving exocyst dots, which colocalize with the motor chimera. Note other non-moving signal also colocalize. A kymograph along the path of the indicated dot is shown on the right. Average rate of movement is shown on the right. Time is indicated on top in seconds. All images are single widefield images. Bar is 2 µm.

Exocyst localization to the cell tips does not require transport along actin cables. However, Sec3, Sec5 and Exo70 were found to travel toward cell tips as discreet dots at speeds of about 1.2 µm/sec (Figure 5A). This speed corresponds to that reported for Myo52 suggesting that exocyst subunits are transported by this type-V myosin [42], [43]. Myo52-dependent movements of Sec6 and Sec8 exocyst subunits were also previously suggested [31]. Consistent with this we were unable to detect tip-directed dots when Sec3-, Sec5- or Exo70-GFP fusions were imaged in a *myo52*Δ background (Figure 5B).

To test more directly if Myo52 can transport the exocyst we made use of our recently described kinesin-myosin chimera, in which the motor domain of Myo52 is replaced by that of kinesin Tea2 (Tea2^N^) and tagged with CFP [44]. This Tea2^N^-CFP-Myo52^C^ chimeric motor transports Myo52 cargos along microtubules and restores rod-shape to *myo52*Δ cells. Because kinesins move much slower than myosins, this approach has the benefit of reducing Myo52 cargo speed thus allowing for the co-localization of the kinesin chimera and potential cargo as they travel along microtubules. In *myo52*Δ cells expressing the Tea2^N^-CFP-Myo52^C^ chimera, Sec3-, Sec5- and Exo70-GFP dots moved towards the cell tips together with the chimera at about 2.2 µm/min (Figure 5C). In addition, exocyst subunits and the kinesin-myosin chimera co-localized extensively at static sites, likely representing aggregated Myo52C-exocyst vesicles not actively transported by the kinesin motor. These data indicate that Myo52 transports exocyst subunits, including Sec3, to cell tips.

## Discussion

Polarized exocytosis is fundamental for many cellular processes. The exocyst plays a critical role in transmitting the spatial information determined by Rho polarity regulators by tethering secretory vesicles for polarized exocytosis [45]. Here we report the identification and characterization of fission yeast Sec3. Collectively our data indicate that Sec3 and Exo70 are redundantly required for the localization of all other exocyst subunits to sites of growth, with Sec3 playing the most important role. However, in fission yeast Sec3 and Exo70 are not pre-localized landmarks at sites of growth as receivers for the other subunits. In contrast, Sec3 and Exo70, together with all exocyst subunits, are co-transported with vesicles by type V myosins to sites of growth. This suggests the whole exocyst complex is already assembled on vesicles.

### Divergent Structure and Function of Fission Yeast Sec3

While previous attempts to identify Sec3 in fission yeast by amino acid sequence similarity were unsuccessful, the identification of a cryptic PH domain in the budding yeast Sec3 N-terminal domain provided a basis for sequence analysis [23], [24]. Because the binding of PIP_2_ is essential for exocyst function in budding yeast, we hypothesized that the missing Sec3 protein would contain a similar cryptic PH domain. This cryptic PH domain on fission yeast Sec3 is likely functional for phospholipid binding as judged by similarity and conservation of charged residues likely to contact PIP_2_ head group phosphates. Additionally, cell tip localization of Sec3-GFP was found to be dependent on the major kinase responsible for production of PIP_2_ (Figure 4A). For budding yeast this domain has also been found to co-crystalize with Rho1, further suggesting the protein-protein interaction interface with Rho proteins is conserved [23]. However, the similarity with budding yeast Sec3 ends with this domain and the C-terminal portion of fission yeast Sec3 shows little similarity to budding yeast. Sequence prediction failed to identify a putative coiled coil region adjacent the PH domain. Instead, a possible coiled coil domain is predicted at the extreme C terminal region of Sec3 preceded by predicted unstructured sequences (Figure 1A). It will be interesting to determine the specificity of this non-conserved part of Sec3 in defining the architecture of the exocyst holocomplex.

### Conditional Essentiality of Sec3 and Exo70

Several lines of evidence, in addition to sequence analysis, support the notion that the gene identified here represents the missing fission yeast *sec3* gene. First, *sec3* mutants display the characteristic multi-septated phenotype of other exocyst mutants. Second, *sec3* mutants are deficient in secretion. Third, *sec3* mutants show specific genetic interaction with *exo70*Δ.

Perhaps surprisingly, *sec3* deletion, like *exo70*Δ, is only conditionally lethal: although multi-septated, *sec3*Δ cells can live when grown in presence of an osmotic stabilizer. How does osmotic stabilization permit life with a compromised exocyst complex? Osmotic stabilization leads to a reduction in turgor pressure inside the cell. This leads to slower growth – the generation time in presence of 1 M sorbitol is about 6 h compared to 4 h in minimal growth medium at 25°C – and reduces the forces required for membrane remodeling events, such as endocytosis [46], [47]. Thus the membrane may be under lower tension than upon normal turgor pressure, increasing the time for and probability of fusion of poorly tethered vesicles.

Even with osmotic stabilization, *sec3*Δ becomes essential in absence of *exo70*Δ. This suggests *sec3* and *exo70* are acting redundantly. In contrast, all other exocyst subunits tested are un-conditionally essential. This is similar to the redundant function of Sec3 and Exo70 PIP_2_ and Rho-binding domains in exocyst localization in the budding yeast [48]. Our localization dependency tests are also in agreement with this model. First, Sec3 and Exo70 localize to cell poles independently of one another. Second, other exocyst subunits failed to localize to cell poles in *sec3 exo70* double mutants, but not in single mutants. We conclude that Sec3 and Exo70 redundantly serve to localize the exocyst at sites of polarization.

In the *sec3-2 exo70*Δ double mutant at restrictive temperature other exocyst subunits formed aggregates in the cytoplasm. We also observed that F-actin (as labeled by phalloidin staining) and myosin V Myo52 displayed similar aberrant localization in these conditions (data not shown). We hypothesize that in absence of functional tethering subunits, the remainder of the exocyst complex does not dissociate and remains bound to vesicles and Myo52, which may aggregate together. This may also in turn affect actin organization.

### Myosin-dependent and -independent Localization of Sec3 and Exocyst Subunits

One important question is how Sec3 and Exo70 and the rest of the complex get localized to cell poles. Our FRAP experiments extend our previous results [32] that actin cables and more generally the actin cytoskeleton are not essential for the localization of any exocyst subunit at cell poles. This is in agreement with the fact that actin-based transport is not essential for polarized cell growth in fission yeast.

However, we now also show that all exocyst subunits, including Sec3 and Exo70, are in fact transported by myosin V. The case for Sec3 and Exo70 as Myo52 cargos is strong: the movement of exocyst subunits towards cell tips occurs at the same rate as Myo52 tail, whether this is linked to the endogenous myosin motor or to Tea2 kinesin motor. This assay is very similar to what has been argued is the gold-standard test for class V myosin-dependent cargo transport: corresponding speed reduction of cargo in step-size mutants of the class V myosin [49]. Thus, even though actin-based transport is not essential, Sec3 is transported along with the rest of the complex along actin cables.

**Figure 6:**
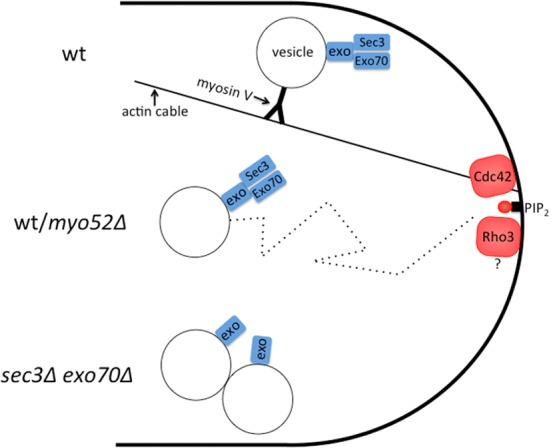
Schematic model suggesting the presence of the entire exocyst complex on exocytic vesicles. Exocytic vesicles labeled with the exocyst holocomplex may reach cell poles through one of two alternative routes: they are either transported along actin cables by myosin V Myo52 or reach cell poles by random walk. At the cell poles, Sec3 and Exo70 tether the exocyst complex and the vesicle by binding PIP_2_ and Rho proteins. In absence of myosin V transport (*myo52*Δ), vesicles and the entire exocyst reach the pole by random walk. In absence of both Sec3 and Exo70, vesicles and the rest of the exocyst complex fail to be tethered at cell poles and form aggregates. Exo represents Sec5, Sec6, Sec8, Sec10, Sec15 and Exo84.

We suggest that these two observations are not contradictory. In fission yeast, secretory vesicles can reach cell poles by two distinct routes – either through myosin-V directed transport or by random walk and capture at the membrane. Thus the simplest model to account for our data proposes that the whole exocyst complex assembles on vesicles, rendering them fusion competent as they reach cell poles through either of the two routes (Figure 6).

### Distinct Requirements of Myosin-based Transport in Fission and Budding Yeasts

This model departs slightly from that proposed in the budding yeast, where most, but not all the exocyst complex is thought to assemble on vesicles. There, the prevalent view is that Sec3 localizes to sites of growth independently of vesicles and acts as pre-localized landmark for incoming vesicles [28], [48], [50], [51], [52]. In contrast, all other subunits are strictly dependent on actin-based transport for delivery to the bud (with Exo70 showing mixed behavior) [8], [28], [51], [52], [53]. Thus, Sec3 and other subunits display distinct modes of localization. In contrast, we show that fission yeast exocyst subunits appear to all have the same localization requirements.

The difference in myosin-based transport requirement may be due to the distinct geometries of the cells: While random walk and capture may be efficient enough in the fission yeast, the bud neck of *S. cerevisiae* may impose a geometrical constrain limiting diffusion, which can only be overcome by directed transport. The difference observed in Sec3 transport may be more minor than appears at first glance. For instance, possible directional Sec3 movements have to our knowledge not been investigated in budding yeast. In addition, immunostaining studies, in contrast to GFP-tagging analyses, have shown delocalized or partly delocalized Sec3 upon actin disruption, suggesting that at least some pools of Sec3 may be dependent on actin-based transport [48], [50]. Similarly, a mutant budding yeast Sec3ΔN allele that lacks the PIP_2_ and Rho-binding domain requires actin cables to localize to the bud [48], indicating budding yeast Sec3 has the ability to be transported along with the rest of the complex. One possibility is that the Sec3 PH-like domain has distinct affinities for PIP_2_ and Rho proteins in the two organisms: strong affinity in budding yeast may tip the balance towards plasma membrane binding at the bud tip, while weaker affinity may promote exocyst holocomplex formation on vesicles in fission yeast.

If the whole exocyst complex assembles on vesicles and Sec3 does not act as pre-localized landmark, how are sites of polarized exocytosis marked? As PIP_2_ and Rho-binding domains are conserved on both Sec3 and Exo70, PIP_2_ and Rho proteins likely act as spatial determinants in all species. Consistently, Exo70 has been shown to bind Cdc42 in fission yeast [54]. Sec3 and Exo70 may then function as coincidence detectors of PIP_2_ and Rho proteins at the plasma membrane, as previously proposed [24]. This is in agreement with our findings that in fission yeast the localization of Sec3, like that of other exocyst subunits, requires PIP_2_ and a functional Cdc42. In addition to Cdc42, Rho3 may also contribute to the localization of these proteins, as *rho3*Δ is synthetic lethal with *exo70*Δ [38]. In summary, our analysis of fission yeast Sec3 extends the notion that Sec3 and Exo70 act as localization determinants in all species examined to date, with the twist that, at least in some cell types, these subunits may label specific cargos, rather than specific target membranes.

## Materials and Methods

### Strains, Growth Conditions and Pharmacological Inhibitor

*S. pombe* strains used in this study are listed in Table 1. Cells were routinely grown in either Edinburgh minimal media (EMM) supplemented with appropriate supplements (ALU) or YE5S at 25°C. When indicated liquid cultures or plates were incubated at 36°C. Strains encoding the *sec3*Δ and *sec3-2* mutations were grown in EMM 1 M sorbitol at 25°C and shifted to EMM without sorbitol or YE5S by washing 3 times in each respective media before re-suspension. Tetrads were germinated on YE5S with and without 1 M sorbitol and incubated at 25°C. Latrunculin A in DMSO (Phillip Crews) was used at a final concentration of 200 µM by dilution of a 100× stock.

**Table 1:** Strains used in this study.

YSM1180	*h- ade6-M210 leu1-32 ura4-D18*	Lab strain
YSM1526	*sec8::ura4+/sec8-13myc-kanMX ade6-M210/ade6-M216 ura4D-18/ura4-D18 leu1-32/leu1-32*	[29]
YSM1568	*h+ sec8-1 leu1-32 ura4-D18*	Lab strain
YSM1742	*h+ exo70::kanMX ade6- leu1-32 ura4-D18*	[32]
YSM1821	*h+/h- sec6::nat/sec6+ ade6-M210/ade6-M216 ura4D-18/ura4-D18 leu1-32/leu1-32*	[32]
YSM2067	*h+/h- sec3+/sec3::kanMX ade6-M210/ade6-M216 ura4D-18/ura4-D18 leu1-32/leu1-32*	This work
YSM2068	*h- sec3-GFP-kanMX ade6-M210 leu1-32 ura4-D18*	This work
YSM2069	*h- sec3-his5Δc+ura4+ his5*Δ*1 ura4-D18*	This work
YSM2070	*h- sec3-2-his5+-ura4+ ade6- leu1- ura4-D18*	This work
YSM2071	*h- sec3-4-his5+-ura4+ his5*Δ*1 ade6+ leu1+ ura4-D18*	This work
YSM2072	*sec3::kanMX ade6- leu1-32 ura4-D18*	This work
YSM2073	*sec3-2-his5+-ura4+ exo70::natMX ura4-D18*	This work
YSM2074	h+/h- sec3+/sec3::kanMX exo70+/exo70::natMX ade6-M210/ade6-M216 ura4D-18/ura4-D18 leu1-32/leu1-32	This work
YSM2095	*h- sec5-GFP-kanMX sec3::kanMX ade6-M210 leu1-32 ura4-D18*	This work
YSM2075	*h- exo70-GFP-kanMX ade6-M210 leu1-32 ura4-D18*	This work
YSM2076	*h- exo70-GFP-kanMX sec3::kanMX ade6-M210 leu1-32 ura4-D18*	This work
YSM2077	*sec3-GFP-kanMX exo70::natMX ade6- leu1-32 ura4-D18*	This work
YSM2078	*h- sec5-GFP-kanMX ade6-M210 leu1-32 ura4-D18*	This work
YSM2079	*h+ sec5-GFP-kanMX exo70::natMX ade6- leu1-32 ura4-D18*	This work
YSM2080	*h+ sec3-2 his5+-ura4+ sec5-GFP-kanMX ade6-M210 ura4-D18*	This work
YSM2081	*sec3-2 his5+-ura4+ sec5-GFP-kanMX exo70::natMX ura4-D18*	This work
YSM2082	*h- exo84-GFP-kanMx ade6-M210 leu1-32 ura4-D18*	This work
YSM2083	*exo84-GFP-kanMX sec3-2 his5+-ura4+ ura4-D18*	This work
YSM2084	*h- exo70::natMX exo84-GFP-kanMX ade6- leu1-32 ura4-*	This work
YSM2085	*exo70::natMX exo84-GFP-kanMX sec3-2-his5+-ura4+ ade6? leu1? ura4-D18*	This work
YSM2086	*sec3-GFP-kanMX cdc42-1625-kanMX ade6- leu1-32 ura4-D18*	This work
YSM2087	*sec3-GFP-kanMX its3-1 ade6- leu1-32 ura4-D18*	This work
YSM2088	*h- sec15-GFP-kanMX ade6-M210 leu1-32 ura4-D18*	This work
YSM2089	*ura4-294::nmt81-tea2N-CFP-myo52C-ura4+ sec5-GFP-kanMX myo52::nat leu1-32 ura4-D18*	This work
YSM2090	*ura4-294:: nmt81-tea2N-CFP-myo52C-ura4+ sec3-GFP-kanMX myo52::nat leu1-32 ura4-D18*	This work
YSM2091	*ura4-294:: nmt81-tea2N-CFP-myo52C-ura4+ exo70-GFP-kanMX myo52::nat leu1-32 ura4-D18*	This work
YSM2092	*sec3-GFP-kanMX for3::kanMX ade6- leu1-32 ura4-D18*	This work
YSM2093	*sec5-GFP-kanMX for3::kanMX ade6- leu1-32 ura4-D18*	This work
YSM2094	*exo70-GFP-kanMX for3::natMX ade6- leu1-32 ura4-*	This work

For construction of *sec3::kanMX* and *myo52::natMX* mutants, a PCR based approach was used by amplifying each antibiotic cassette from pFA6a-based plasmids with 78 bp extentions with homology directly upstream and downstream the open reading frames. For GFP tagging of exocyst members the GFP-kanMX sequences from pFA6a plasmid was amplified with primers containing 78 bp homology directly upstream and dorwnstream the stop codon. Correct disruption or tagging was confirmed by diagnostic PCR.

### Generation of sec3-2 Temperature Sensitive Lethal Allele

For isolation of the temperature sensitive lethal *sec3-2* allele the marker reconstitution mutagenesis approach described for fission yeast was used [55]. This approach involves a two-step procedure, which requires the construction of a strain containing a portion of the *his5* selection marker downstream a gene of interest. This is then followed by the site-specific recombination of mutagenic PCR of the gene of interest containing the remaining sequences of the *his5* marker. For construction of the recipient strain a plasmid was first constructed which encodes the His5N domain and *ura4+* flanked by about 500 bp 3′ sequences of *sec3* and about 500 bp 3′ UTR sequences of *sec3*. First, two PCR reactions of gDNA template and primers osm1310 (gctGGATCCgcatttgtatcatctacggc) and osm1314 (gtttctctGGCGCGCCgggcaactccttcgactgg) and osm1309 (gttgcccGGCGCGCCagagaaacgctcggcttctg) and osm1311 (cagGTCGACgcttcacgacaaatatcgaaag) were isolated. These two products were stitched together by PCR using primers osm1310 and osm1311. The resultant 1109 bp product was then digested with BamHI and SalI and ligated to similarly treated pH5ΔcU4+ [55] yielding plasmid pSM1007. This plasmid was digested with AscI yielding linear the linear plasmid containing *sec3(3′)-his5N-ura4+-sec3(3′UTR)*. The linear DNA was transformed into strain YSM1867 yielding strain YVV40.

The plasmid template for mutagenic PCR was made as follows. PCR of gDNA template and primers osm1329 (cagGTCGACcccttgcttataaagactc) and osm1336 (gctGGATCCctttcgatatttgtcgtgaagc) (2067 bp) was digested with SalI and BamHI and ligated to similarly treated pH5c+ [55]. The resulting plasmid pSM1007 (*sec3-his5C)* was used as template for mutagenic PCR with primers osm1272 (cccttgcttataaagactc) and osm1374 (gacgaagctctttctagaagcgtagt). Mutagenic PCR was done with Peqlab Taq polymerase and buffer Y modified to 7 mM MgCl_2_. Mutagenic PCR product was transformed into recipient strain YVV40 and plated onto YE. The following day the plates were replica plated onto EMM lacking histidine and allowed to form colonies. Temperature sensitive mutants were screened at 36°C. Sequencing of the *sec3-2* allele revealed a point mutation at codon 577 of TGT to CGT resulting in amino acid substitution C577R. Sequencing of *sec3-4* revealed the same mutation as *sec3-2*.

### Microscopy

Microscopy was performed by either spinning disk confocal or widefield microscopy. Spinning disk microscopy was carried out using a Leica DMI4000B inverted microscope equipped with an HCX PL APO ×100/1.46 numerical aperture (NA) oil objective and a PerkinElmer Confocal system (including a Yokagawa CSU22 real-time confocal scanning head, and solid-state laser lines. Stacks of *z*-series confocal sections were acquired at 0.3-µm intervals using Volocity software. Unless otherwise indicated, images shown are two-dimensional maximum-intensity projections. Widefield microscopy was performed on a DeltaVision platform (Applied Precision) composed of a customized Olympus IX-71 inverted microscope and a UPlan Apo 100X/1.4 NA oil objective, a CoolSNAP HQ2 camera (Photometrics), and an Insight SSI 7 color combined unit illuminator. Unless otherwise indicated, images shown are maximum projections of deconvolved z-slices taken every 0.3 µm. Figures were prepared with Image J v1.46 and Photoshop CS5.

Imaging of cells at 36°C (Figure 3B) was performed with an objective heater and Delta T specimen warming dish from Bioptech (Butler, PA). Cells were grown overnight in EMM 1 M sorbitol concentrated by centrifugation and adhered to Delta T dishes with equal volume of 100 µg/ml lectin (Bandeiraea simplicifolia, Sigma). Cells were then washed with and grown in EMM. Cells were imaged 90 minutes after shift to 36°C. All other imaging was done at room temperature (about 23°C).

Imaging of the Tea2^N^-CFP-Myo52^C^ chimera and exocyst-GFP fusion was done as follows. Precultures in EMM supplemented with thiamine were washed 3 times in EMM and grown for 18 hours in EMM at 25°C. Cells were placed onto 2% agarose EMM pads and covered with a slide and sealed with VALAP prior to imaging. Images of CFP and YFP signals were acquired every 10 seconds for 3 minutes. Control imaging of cells expressing only the Tea2^N^-CFP-Myo52^C^ chimera confirmed the GFP signal captured in the YFP channel was not due to bleed-through of CFP fluorescence.

### Fluorescence Recovery after Photobleaching (FRAP)

FRAP experiments were performed with the Photokinesis module attachment for the spinning disk confocal microscope (PerkinElmer). Cells were placed onto 2% agarose EMM pads and covered with a slide and sealed with VALAP prior to imaging. For cells treated with LatA and DMSO, the drug or solvent was added to melted agarose prior to pad construction and imaged within 5 minutes. Photobleaching was carried out by applying 488-nm laser to cell tips. Images were acquired before photobleaching, immediately after, and subsequently at regular intervals. Analysis of FRAP was done as described previously [56]. For each individual FRAP curve, the mobile fraction was estimated by averaging values for time points between 35 and 50 seconds after photobleaching. The halftime was given by the time at which the half-maximal value was reached. In Figure 4C, plotted values were normalized to the average maximum recovery for each strain.

### Measurement of Acid Phosphatase Secretion

Acid phosphatase secretion was measured as described previously with modifications [32]. Precultures (6 ml) of cells were grown overnight in EMM 1 M sorbitol at 25°C. Cells were then diluted to OD_600_ = 0.05 or 0.1 (YSM2072 and YSM2073 in 75 ml EMM 1 M sorbitol and incubated overnight at 25°C. Cells were then washed once in either EMM with or without 1 M sorbitol and resuspended to OD_600_ = 0.3 and grown at either 25°C or 36°C. At each time point 500 µl aliquots were removed and centrifuged, and 350 µl of the supernatant was added to 350 µl of 2 mM p-nitrophenyl phosphate 0.1 M sodium acetate, pH 4.1, prewarmed to 30°C. Reactions were incubated at 30°C for 10 min and then stopped by the addition of 350 µl 1 M NaOH. Acid phosphatase acitivity was determined by measuring the absorbance at 405 nm and normalized by subtracting the values at time point 0. Values were then normalized to the OD_600_ readings for each time point.
